# Chronic Lumbar Pain and Insomnia in College-Aged Students

**DOI:** 10.3390/healthcare10040701

**Published:** 2022-04-09

**Authors:** Katsumi Hamaoka, Ryouta Ashizawa, Mitsumasa Hida, Ippei Suganuma, Yoshinobu Yoshimoto

**Affiliations:** 1 Department of Physical Therapy, Faculty of Health Science, Osaka Yukioka College of Health Science, Osaka 567-0801, Japan; 2 Rehabilitation Department, Seirei Mikatahara Hospital, Shizuoka 433-8558, Japan; 19dr01@g.seirei.ac.jp; 3 School of Rehabilitation, Osaka Kawasaki Rehabilitation University, Osaka 597-0104, Japan; pthida.mitsum@gmail.com; 4 Department of Occupational Therapy, Faculty of Health and Medical Sciences, Yamato University, Osaka 564-0082, Japan; suganuma.ippei@yamato-u.ac.jp; 5 Department of Physical Therapy, Seirei Christopher University, Shizuoka 433-8558, Japan; yoshinobu-y@seirei.ac.jp

**Keywords:** insomnia, college student, chronic lower back pain

## Abstract

Insomnia in college students has a significant impact on academic performance and mental health (e.g., depression). Although the mechanisms underlying insomnia and chronic pain are becoming clearer, only a few studies on college students have examined these factors by their location in the body. The purpose of the present study was to identify the location of chronic pain in the body most associated with insomnia in college students. A web-based survey was used to collect information pertaining to nine questions from 494 university students: sex, age, presence of chronic pain, intensity of chronic pain, location of chronic pain, and duration of chronic pain, as well as scores from the Athens Insomnia Scale (AIS), Pain Catastrophizing Scale, and Hospital Anxiety and Depression Scale. To examine the association between insomnia and the site of chronic pain, stepwise logistic regression analysis was conducted with AIS as the target variable. The results showed a significant positive correlation between chronic pain in the lumbar region and AIS scores. Future longitudinal studies including multiple factors are necessary to clarify the causal relationship between insomnia and chronic lower back pain.

## 1. Introduction

The prevalence of insomnia among college students is high (40–70%) [1]. College students may undergo rapid lifestyle changes that increase stress levels, such as building relationships and learning new academic subjects [2]. Accordingly, it is important to address insomnia in college students, as it has been reported to affect academic performance [3,4] and mental health, including causing depression [5,6]. Various factors have been reported to contribute to the development of insomnia, including age, sex, and comorbidities; however, such factors are relatively difficult to control. Alternatively, chronic pain has been found to positively correlate with insomnia in college students [7] and has attracted attention as a controllable factor [8,9,10]. Palermo et al. [11] reported that insomnia and chronic pain were closely related and that > 40% of subjects with insomnia maintained ≥ 1 chronic pain location in the body. Previous studies on the elderly have also reported that chronic pain is a predictor of insomnia [12].

The positive correlations between chronic pain and insomnia are logical, as pain can make it difficult to fall asleep or shorten sleep duration. Chronic pain affects sleep quality as well as sleep duration. Poor sleep quality can result in a negative feedback loop that further exacerbates chronic pain as sleep quality is further affected [13,14]. In addition, long-term insomnia exacerbates anxiety, depression, and other symptoms and significantly reduces the quality of life [15,16].

Recommended methods to reduce chronic pain include exercise therapy [17] and medication therapy [18]. However, college students are less active [19], suggesting that they are less likely to implement and continue exercise therapy. On the other hand, medication therapy is used to improve insomnia but increases the risk of medication dependence [20].

Previous studies investigating chronic pain and sleep quality reported that more than half of the subjects complained of sleep fragmentation, with exacerbated pain, waking more than three times during sleep, sleeping less than five hours, and requiring more than 30 min to fall asleep [21].

However, most previous studies were conducted on the elderly [22,23], and of the few that have investigated the relationship between insomnia and chronic pain in college students, none have been sufficiently validated. As a large percentage of university students experience chronic pain [24] and since insomnia is likely to affect their academic performance, including participation in lectures [3,4], there is a significant need to clarify this relationship.

It remains unclear whether chronic pain in certain parts of the body is more closely associated with insomnia. Numerous studies have reported that the presence, intensity, and number of chronic pain events are associated with insomnia [11,25,26]; however, there is still a need for a detailed evaluation of chronic pain intensity and body sites of these events. Earlier studies investigating the location of chronic pain throughout the body of elderly patients [27,28] revealed that 53.5% of patients experience pain events in the lower limbs and feet, 23.6% in the head, 21.1% in the abdomen and pelvis, 19.1% in the neck and shoulder joints, 18.8% in the forearm and wrist joints, 15.7% in the chest, 14.4% in the lower back, and 3.3% in the face. Accordingly, there is a need to clarify the presence, intensity, and bodily location of chronic pain to potentially prevent or alleviate insomnia; however, to the best of the authors’ knowledge, no such studies have investigated the relationship between the location of chronic pain in the body and insomnia among college students.

To this end, the purpose of the present study was to identify the sites of chronic pain most closely associated with insomnia in college students.

## 2. Materials and Methods

The subjects of this study were college students 18 years of age or older who were affiliated with Yamato University. A cross-sectional study was conducted using the following exclusion criteria: non-university students, those with pre-existing psychiatric disorders, and those who required any form of assistance in their daily lives, those who were unable to provide or withdrew their consent for the study, and those who withdrew from their university.

Recruitment methods for this study included invitations to participate in the study from research collaborators and the distribution of posters.

The web-based survey research methods used here have recently been employed in medical publications and large-scale studies; further, they represent a surveying technique that does not differ significantly from postal methods [29]. The web survey included nine questions: age; gender; existence, intensity, duration, and bodily location of chronic pain; as well as scores from the Athens Insomnia Scale (AIS), Pain Catastrophizing Scale (PCS), and Hospital Anxiety and Depression Scale (HADS).

Assessment of insomnia via the AIS required a questionnaire survey of eight sleep-related questions. Each question was scored on a four-point scale (0–3), and the total was used to determine the degree of insomnia. The AIS results can ultimately be divided into the following categories: “No problem of insomnia”, total score of 0–3; “Slight suspicion of insomnia”, total score of 4–5; “Suspicion of insomnia”, total score of 6–9; and “Recommend consultation with a specialist”, total score of ≥10 [30,31,32]. In accordance with previous research, subjects were divided into two groups: those with insomnia (total AIS score ≥ 6), and those without insomnia (AIS < 6) [31].

Here, chronic pain was defined as “persistent or recurrent pain for >3 months” [33]. The Brief Pain Inventory [34] was used to further investigate chronic pain, where a numerical rating scale (NRS) is used to assess the intensity of chronic pain [35]. The Brief Pain Inventory is a numerical evaluation of the degree of pain, mood, and behavior disturbed by pain on a scale of 0 to 10, with 0 representing no pain at all and 10 representing the most severe pain. Higher scores indicate more pain and impaired mood and behavior.

The investigation into the bodily location of chronic pain used multiple-choice analyses, where body parts were checked off using an anatomical diagram [34]. Subsequently, the selected responses were classified into the following categories: head, neck, shoulder, upper arm, elbow, forearm, wrist, fingers, back, lumbar, chest, abdomen, hip, thigh, knee, lower leg, ankle, and toes. Each body part was coded numerically from 1 to 18.

The PCS is a 13-part questionnaire containing five responses for assessment of catastrophizing pain, rumination, helplessness, and magnified vision. Scores range from 0 to 52 points, where ≥30 is judged to be catastrophic for pain [36].

The HADS is a 14-part questionnaire consisting of seven questions related to each of depression and anxiety over the past week [37]. The four-point scale yielded a total score range from 0 to 42, where higher scores pertained to greater levels of anxiety and depression. Here, HADS values were divided accordingly: 0–7 points referred to “no anxiety or depression”, 8–10 points were deemed “suspicious”, and ≥11 points were considered “anxious or depressed”.

To statistically examine the association between the presence of insomnia and location of chronic pain, stepwise logistic regression analyses were conducted using the presence of insomnia as the target variable, and the existence, strength, duration, and locations of chronic pain, as well as covariates (sex, age, PCS, and HADS scores) as the explanatory variables. The statistical significance level was set at α = 0.05. The statistical significance level was set at 5%. When a correlation coefficient of 0.9 or higher was found between two covariates, one of the covariates was removed to account for multicollinearity.

## 3. Results

Of the 524 original subjects, 494 were included in the final analysis following the application of exclusion criteria (186 males, 308 females, mean age 18.6 ± 1.1 year). The results showed that 86 (17.4%) college students had insomnia whereas 148 (30.0%) had chronic pain. The average HADS and PCS scores were 11.71 and 18.82, respectively (Table 1). Among those experiencing chronic pain, the lumbar region was the most common bodily location (n = 72, 48.6%), followed by 43 (29.1%) experiencing pain in the shoulder joints and 35 (23.6%) experiencing pain in the head (Table 2). 

The results of the stepwise logistic regression analyses with insomnia as the objective variable and chronic head, neck, shoulder, lumbar, hip, knee, NRS, gender, age, PCS, and HADS as explanatory variables showed a significant positive correlation between chronic pain in the lumbar region and insomnia (Table 3).

## 4. Discussion

The results of our study revealed that insomnia in college students was positively correlated with chronic lower back pain. A previous study found that chronic lower back pain was correlated with decreased sleep quality, increased time spent asleep, time required to fall asleep, decreased daytime functioning, and unsatisfactory sleep [38]. Other studies have shown that subjects with chronic lower back pain have a greater frequency of sleep disorders [39]. Additional studies have also reported a significant correlation between chronic lower back pain and sleep [25,26,40], corroborating the trends revealed in the current study.

Numerous mechanisms by which chronic lower back pain can induce insomnia exist. Chronic pain (not only chronic lower back pain) has been shown to impact both physical and psychological health as it is likely to be accompanied by decreased movement [41] and walking motion [42], along with higher rates of depression [41,43]. During bouts of lower back pain, the sympathetic nervous system is activated [44], while the continuity of chronic lower back pain can lead to its overactivity, thereby increasing the concentration of inflammatory cytokines in the body [45,46] in a process known to be associated with sleep disorders [47]. Additionally, college students’ weekly sitting time is >30 h [48]. Excessive time spent in a seated position has a known burden on health [49], leading to both physical and mental discomfort [50]. The sitting posture deforms the lumbar spine and increases the internal pressure on the lumbar region, both of which are amplified during prolonged sitting [51]. Accordingly, college students are continuously subjected to mechanical stress on their lower back and are thus more likely to experience chronic back pain. These mechanisms are consistent with the results of the present study, thereby suggesting the root of chronic lumbar pain among the studied sample. 

Psychosomatic interventions such as acupuncture [52], cognitive behavioral therapy, yoga, and tai chi [53] have been reported to be effective for the treatment of chronic lower back pain. It has been suggested that interventions for chronic low back pain such as acupuncture, moxibustion, and cognitive behavioral therapy aid in alleviating insomnia.

The strength of the present study lies in its assessment of insomnia as related to body location of chronic pain, whereas conventional studies have grouped all such chronic symptoms into a single category [11,25,26], inhibiting the ability to use probability-specific measures for reducing chronic pain. By categorizing the bodily regions of chronic pain, and further examining insomnia as it relates to these locations, the precise relationship between chronic pain in each body area can be clarified, greatly aiding the implementation of any countermeasures.

Furthermore, the size of this survey study (approximately 500 university students) improves the reliability of the statistical analyses and thus represents the first known comprehensive study of insomnia among college students.

There are, however, several limitations in the present study. First, the causal relationship between insomnia and chronic lower back pain remains unknown. As this was a cross-sectional study, the directionality of this relationship was not clarified, something that should be addressed in further prospective cohort studies. Second, investigation of lifestyle habits that are thought to affect insomnia, as well as data collection regarding biological changes, were limited. Because lifestyle changes affect physical and mental stress [54], it is necessary to quantitatively incorporate the effects of lifestyle and stress in future analyses. Additionally, there are several types of insomnia, such as trouble falling asleep and mid-wakefulness. In this study, the AIS was used, and it was difficult to classify insomnia by type. It was suggested that future studies are needed to evaluate the possibility of classifying insomnia by type and the location of chronic pain. Insomnia is greatly influenced by lifestyle. In this study, it was difficult to interview college students about their lifestyles. It was suggested that there is a need to examine the effect of lifestyle on insomnia in the future.

Here, insomnia in college students was found to be positively correlated with chronic lower back pain. Accordingly, to help prevent or alleviate insomnia in college students, there is a strong need for a functional lifestyle approach while focusing particularly on chronic lower back pain.

## Figures and Tables

**Table 1:** Research subject attributes and questionnaire survey results: AIS—Athens Insomnia Scale; HADS—Hospital Anxiety and Depression Scale; PCS—Pain Catastrophizing Scale.

Variable	Number	Rate (%)	Mean ± SD
Female	308	62.3	
Male	186	37.7	
AIS total score			3.32 ± 2.946
Insomnia	86	17.4	8.28 ± 2.798
No insomnia	408	82.6	2.28 ± 1.609
Chronic pain	148	30.0	
No chronic pain	346	70.0	
HADS			11.71 ± 5.727
Anxiety	161	32.6	17.15 ± 4.76
No anxiety	333	67.4	9.08 ± 4.06
Depression	126	25.5	17.37 ± 5.02
No depression	368	74.5	9.77 ± 4.55
PCS			18.82 ± 10.857

Anxiety: 8 points or more Depression: 8 points or more.

**Table 2:** Results of the site of chronic pain in the study subjects.

Location of Chronic Pain	Number	Ratio (%)
Lumbar	72	48.6
Shoulder joint	43	29.1
Head	35	23.6
Neck	29	19.6
Knee joint	30	20.3
Ankle joint	23	15.5
Back	19	12.8
Abdomen	19	12.8
Hip Joint	10	6.8
Chest	9	6.1
Elbow joint	7	4.7
Hand and finger	7	4.7
Upper limbs	5	3.4
Hand joint	5	3.4
Toe	0	0
Other	5	3.4
Total	318	

Duplicate answers were given for chronic pain sites.

**Table 3:** Logistic regression analyses results.

Variable	OR [95% CI]	*p*-Value
Lower back	2.284 [2.284–1.275]	<0.007
HADS total score	1.203 [1.145–1.264]	<0.000
PCS total score	1.029 [1.004–1.055]	<0.021

Model χ^2^: 89.662 Cox–Snell R^2^: 0.166. Nagelkerke R^2^: 0.275 Hosmer–Lemeshow: 0.803.

## Data Availability

Data will be provided to all interested parties upon reasonable request.
